# Conservative Treatment of Duodenal Obstruction Caused by Retroperitoneal Hematoma Following Seatbelt Injury in a Child: A Case Report

**DOI:** 10.1002/ccr3.72313

**Published:** 2026-03-16

**Authors:** Yukina Matsumoto, Sakura Minami, Chikara Watanabe, Daisuke Fujihira, Mafumi Shinohara, Ichiro Takeuchi

**Affiliations:** ^1^ Advanced Critical Care and Emergency Center Yokohama City University Medical Center Yokohama Kanagawa Japan

**Keywords:** abdominal injury, hematoma, pediatrics, retroperitoneal space

## Abstract

Hemodynamically stable children with duodenal obstruction caused by retroperitoneal hematoma after a seat belt injury can be successfully managed conservatively. Careful clinical and imaging monitoring allows nonoperative treatment and may help avoid complications associated with surgical intervention.

## Graphical abstract

A three‐year‐old boy developed duodenal obstruction due to a seatbelt injury‐induced retroperitoneal hematoma. Successful conservative management highlights the importance of careful monitoring and individualized treatment duration.

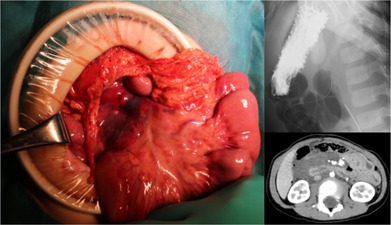

**Abbreviations**
CRP: C‐reactive protein
CT: computed tomography
Hb: hemoglobin
MRI: magnetic resonance imaging
POD: postoperative day
WBC: white blood cell

## 1 Introduction

In Japan, traffic accidents are the leading cause of death among children aged > 2 years [1]. The widespread use of seat belts and child seats has reduced the incidence of severe trauma from ejection during crashes [2]. However, an increase in truncal injuries, particularly those involving the chest and abdomen, due to seat belts, has been noted [2].

Retroperitoneal hematoma is a type of truncal injury; it can occur when a seat belt applies strong pressure to the upper abdomen, causing vascular damage [3]. In children, the supporting tissues around the internal organs are underdeveloped and more fragile than those in adults, and the blood vessels are more susceptible to injury, even with minor external forces [4].

Hematomas often resolve spontaneously depending on the size and location of the hematoma, and conservative management is the standard approach; however, in some cases, hematoma growth and pressure can cause intestinal obstruction [3]. The overall incidence of traumatic retroperitoneal hematoma is difficult to determine, as reported data are heterogeneous and largely derived from adult trauma populations, and the true incidence in children remains unclear and is likely low [5]. Currently, no standardized guidelines exist regarding treatment options, indications for intervention, or surgical approaches in such cases [6]. We report a pediatric case of duodenal obstruction caused by a retroperitoneal hematoma from seat belt trauma that was successfully managed by a conservative approach.

## 2 Case Presentation

**FIGURE 1:**
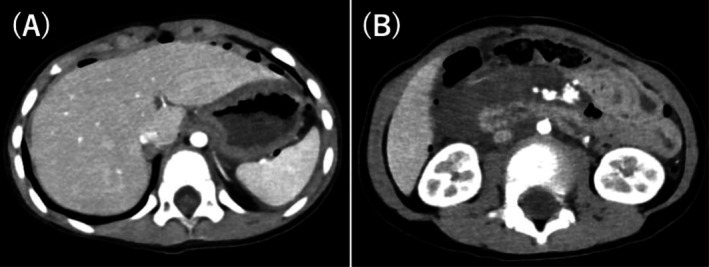
(A) Contrast‐enhanced CT performed at the previous hospital. Free air is observed on the liver surface, suggesting bowel perforation. (B) Contrast‐enhanced CT was performed at the previous hospital. A retroperitoneal hematoma approximately 6 cm in diameter is observed in retroperitoneal Zone 1. No evidence of active extravasation is seen. CT, computed tomography.

A boy aged 3 years and 9 months (height: 102 cm, weight: 16.4 kg) with no significant birth or developmental history was injured in a traffic accident while secured in a child seat in the rear of a car driven by his mother. A contrast‐enhanced computed tomography (CT) scan was performed at the receiving hospital as part of the initial trauma assessment, revealing free air in the abdominal cavity and a hematoma in retroperitoneal zone 1 (Figure 1). The patient was transferred to our hospital for surgical management.

Upon arrival, the patient's vital signs were: respiratory rate: 35/min, SpO_2_: 93% (room air), blood pressure: 124/73 mmHg, heart rate: 146 bpm, Glasgow Coma Scale: E4V5M6, and temperature: 36.6°C.

He was tachycardic and pale, indicating a state of shock, and required continuous intravenous fluid administration. Abdominal examination revealed muscle guarding, and imaging findings from the previous hospital suggested intestinal injury and peritonitis leading to septic shock. Therefore, resuscitation with intravenous fluids was initiated, and due to the urgency of surgical intervention, endotracheal intubation was performed in the resuscitation room.

The patient's blood test results were as follows: white blood cell count: 14,500/μL, hemoglobin: 12.9 g/dL, fibrinogen: 372 mg/dL, D‐dimer: 20.4 μg/mL, amylase: 395 U/L, lipase: 640 U/L, and C‐reactive protein: 0.47 mg/dL.

**FIGURE 2:**
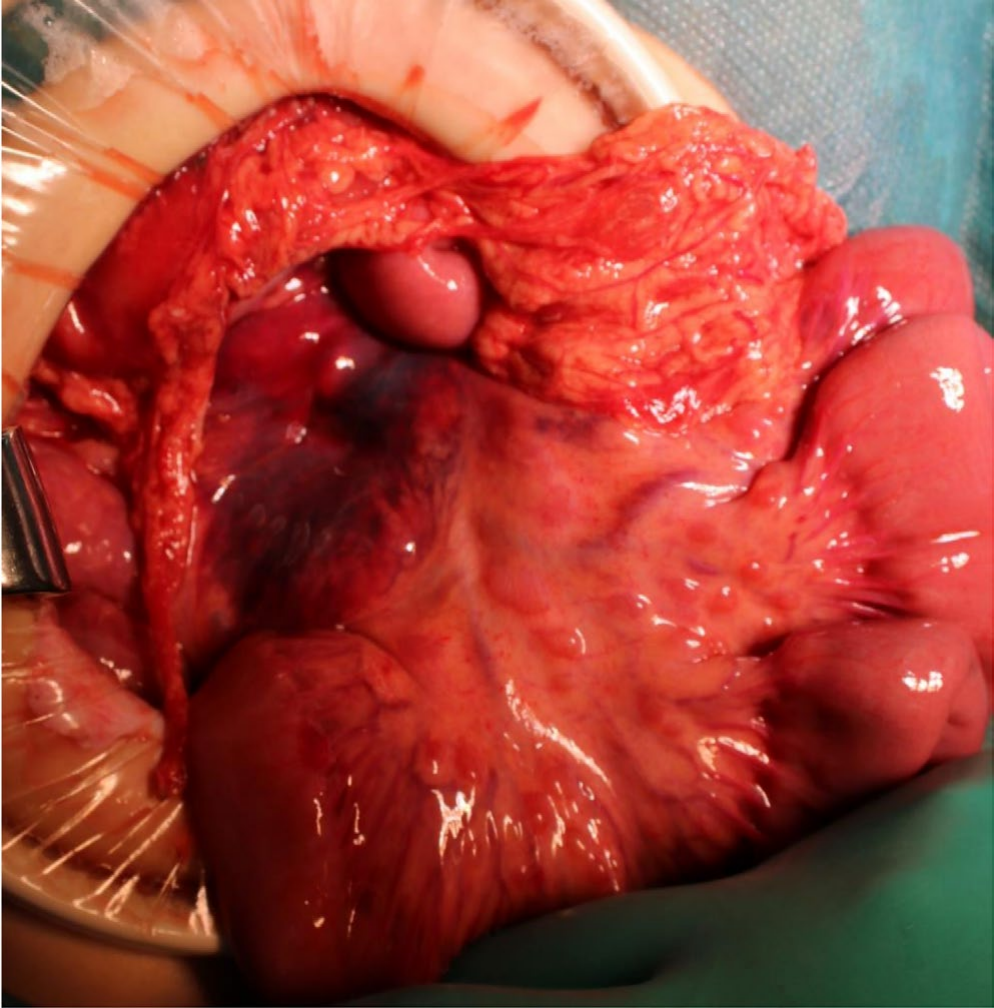
Intraoperative photograph. A large amount of hematoma is observed in the retroperitoneal space.

Emergency laparotomy revealed a 1‐cm perforation in the lesser curvature of the gastric antrum, which was treated with primary closure and omental patching, along with lavage and drainage. A large retroperitoneal hematoma was identified; however, as no active bleeding was noted, it was managed conservatively (Figure 2). The elevated serum amylase level and amylase‐rich drain output suggested pancreatic injury; therefore, magnetic resonance cholangiopancreatography was conducted on postoperative day (POD) 4. No obvious pancreatic duct injury was detected, and conservative treatment was continued, with spontaneous resolution.

**FIGURE 3:**
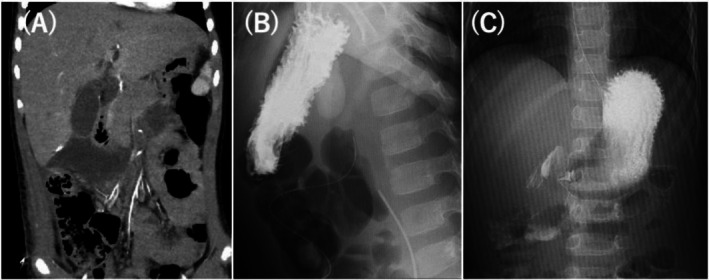
(A) Contrast‐enhanced CT on postoperative day (POD) 7. A hematoma is observed around the duodenum, causing duodenal obstruction. (B) Gastrointestinal contrast study on POD 7 was performed in a right complete lateral decubitus position with the duodenum positioned directly below the stomach; and manual abdominal compression was applied. No contrast medium was seen passing beyond the duodenum. (C) Gastrointestinal contrast study on POD 16. In the supine position, without compression, contrast medium is observed flowing beyond the duodenum. CT, computed tomography.

Despite requiring massive fluid resuscitation for peritonitis, no vasopressor support was needed. On POD 5, hemodynamic stability was achieved, and extubation was performed on POD 7. However, drainage from the gastric tube, initially 5–100 mL/day, increased to 890 mL/day on POD 9, with persistent absence of bowel movements and reduced peristalsis. No bloody or coffee‐ground material was observed in the nasogastric drainage throughout the clinical course. A contrast study and CT were performed to reassess the retroperitional hematoma and exclude associated complications. These studies showed no significant enlargement of the hematoma compared with the initial findings; however, contrast retention was observed in the stomach and duodenal bulb, and duodenal obstruction distal to the second portion (descending part) was suggested, likely due to extrinsic compression from the retroperitoneal hematoma (Figures 3A,B).

Given the high gastric tube output and suspected gastrointestinal transit disorder, enteral nutrition was deemed unfeasible, and total parenteral nutrition was initiated. To avoid frequent radiation exposure in this pediatric patient, gastric tube output was used as a monitoring index. The patient was closely observed for abdominal distension, abdominal compartment syndrome, fever, and inflammatory marker elevation suggestive of hematoma infection.

On POD 16, a contrast study was performed to assess the resolution of duodenal obstruction and readiness for oral intake, which demonstrated contrast passing into the small intestine (Figure 3C), and on POD 19, a non‐contrast CT was performed to confirm the reduction of the retroperitoneal hematoma. With decreasing gastric tube output, oral fluid intake was initiated on the same day. No vomiting or increase in gastric tube output occurred, allowing progression to oral feeding on POD 23. A gradual dietary upgrade was performed, and the patient was discharged home on POD 36. Follow‐up was conducted at the outpatient clinic for 6 months after the surgery, but no abnormalities were found.

## 3 Discussion

In this case, conservative treatment was successful for duodenal obstruction caused by a retroperitoneal hematoma. Although the overall incidence of traumatic retroperitoneal hematoma in children is likely low [5], pediatric patients may be more vulnerable to clinically significant consequences because they have relatively larger abdominal organs and less intra‐abdominal fat than adults [7].

The primary treatment strategies for retroperitoneal hematoma are conservative and surgical. Conservative treatment is applied to hemodynamically stable cases without progressive anemia or severe vascular injury [5, 7]. However, known complications, including bowel obstruction, require careful management [5]. The criteria for conservative management of bowel obstruction include the absence of injuries requiring surgical intervention (e.g., perforation), hemodynamic stability without ongoing bleeding.

Although conservative treatment is less invasive than surgical treatment, it also carries risks. If obstructive symptoms persist or worsen, or if complications such as cholangitis or pancreatitis are suspected, percutaneous drainage or surgical intervention may be required. Furthermore, if there is a risk of intestinal ischemia or perforation, early surgical intervention is necessary [6, 8]. Surgical treatment involves extensive dissection, which may lead to complications, including ureteral injury, intestinal ischemia, or massive bleeding [5]. Therefore, surgical indications should be carefully considered.

There is no established standard regarding the optimal duration of conservative management for traumatic retroperitoneal hematoma causing duodenal obstruction. There are cases involving children, and from what has been published in papers, there are only three cases published, which were operated on within 1–4 days following the occurrence of the injury (Table 1) [6, 8]. With adult individuals, conservative care is more frequently effective, and people are generally able to take by mouth again around 2 weeks after the injury (range: 9–25 days) [9, 10, 11]. When conservative treatment did not succeed, other procedures such as ultrasound‐guided percutaneous (through the skin) drainage or endoscopic balloon dilation were used, and the patients generally recovered clinically within approximately 3–4 weeks [11, 12]. In this context, our case suggests that, even in pediatric patients, conservative management may be a reasonable option when the clinical course is stable and there are no signs of bowel ischemia or perforation.

**TABLE 1:** Summary of reported pediatric cases of duodenal obstruction caused by traumatic retroperitoneal hematoma.

First author, year	Age (year), sex	Cause	Duodenum injury site	Other injury site	Treatment	Duration^a^ (days)
Hirugade [8], 2023	6/M	Trauma	D3, D4	—	Surgery	4
Gue [6], 1972	16/M	Trauma	D3, D4	Transverse colon and Jejunum laceration	Surgery	4
Gue [6], 1972	10/M	Trauma	D2	Duodenal contusion	Surgery	1
Our case	3/M	Trauma	D2	Gastric perforation	Conservative	23

Abbreviations: D2, descending part; D3, horizontal part; D4, ascending part; M, male.

^a^ From injury to start of oral intake in the conservative group, and from injury to surgery in the surgical group.

In this case, conservative treatment was effective for intestinal obstruction caused by a retroperitoneal hematoma. Although the patient developed duodenal obstruction associated with the hematoma, careful monitoring of hemodynamics and nasogastric drainage was performed alongside radiographic assessments, including contrast studies and CT imaging, to observe for any progression requiring surgical intervention. Imaging studies play a crucial role in determining treatment strategies. However, in pediatric patients, radiation exposure must be carefully considered in accordance with the ALARA princaiple [13, 14]. In this case, CT examinations were performed selectively based on clinical deterioration and therapeutic decision‐making. Ultrasonography was limited by bowel gas, and magnetic resonance imaging (MRI) was not selected due to practical limitations in the acute pediatric setting, including examination time and the need for sedation. Ultimately, changes in nasogastric drainage served as a key clinical indicator, and cautious nutritional management with close observation enabled a favorable outcome through conservative treatment.

## 4 Conclusion

In this case, duodenal obstruction caused by a retroperitoneal hematoma was successfully managed without surgical intervention through careful monitoring and appropriate nutritional management. Particularly, close observation based on nasogastric drainage and imaging assessments proved useful. Given the concerns of radiation exposure in pediatric patients, a well‐considered diagnostic strategy is essential. This case suggests the effectiveness of conservative treatment for intestinal obstructions associated with retroperitoneal hematomas.

## Author Contributions

**Yukina Matsumoto:** conceptualization, investigation, writing – original draft. **Sakura Minami:** conceptualization, validation, writing – review and editing. **Chikara Watanabe:** writing – review and editing. **Daisuke Fujihira:** visualization, writing – review and editing. **Mafumi Shinohara:** conceptualization, writing – review and editing. **Ichiro Takeuchi:** supervision.

## Funding

The authors have nothing to report.

## Ethics Statement

Ethical approval is not required at our institution to publish an anonymous case report.

## Consent

Written informed consent for publication of their clinical details and clinical images was obtained from the parents of the patient. Copies of the consent forms are available for review by the Editor of this journal.

## Conflicts of Interest

The authors declare no conflicts of interest.

## Data Availability

The datasets used during the current study are available from the corresponding author on reasonable request.
